# A Comparison of the Association of Fasting Plasma Glucose and HbA1c Levels with Diabetic Retinopathy in Japanese Men

**DOI:** 10.1155/2020/3214676

**Published:** 2020-10-31

**Authors:** Yumi Matsushita, Norio Takeda, Yosuke Nakamura, Natsuyo Yoshida-Hata, Shuichiro Yamamoto, Mitsuhiko Noda, Tetsuji Yokoyama, Tetsuya Mizoue, Toru Nakagawa

**Affiliations:** ^1^Department of Clinical Research, National Center for Global Health and Medicine, 1-21-1 Toyama, Shinjuku-ku, Tokyo 162-8655, Japan; ^2^Takeda Eye Clinic, 2-16-26 Tendai, Inage-ku, Chiba-shi, Chiba 263-0016, Japan; ^3^Department of Ophthalmology and Visual Science, Chiba University Graduate School of Medicine, 1-8-1 Inohana, Chuo-ku, Chiba 260-8670, Japan; ^4^Department of Ophthalmology, Tokyo Hospital, 3-15-2 Ekota, Nakano-ku, Tokyo 165-8906, Japan; ^5^Hitachi, Ltd. Hitachi Health Care Center, 4-3-16, Ose-cho, Hitachi-shi, Ibaraki 317-0076, Japan; ^6^Department of Diabetes, Metabolism and Endocrinology, Ichikawa Hospital, International University of Health and Welfare, 6-1-14 Kounodai, Ichikawa, Chiba 272-0827, Japan; ^7^Department of Health Promotion, National Institute of Public Health, 2-3-6 Minami, Wako, Saitama 351-0197, Japan; ^8^Department of Epidemiology and Prevention, National Center for Global Health and Medicine, 1-21-1 Toyama, Shinjuku-ku, Tokyo 162-8655, Japan

## Abstract

**Introduction:**

The relationship between HbA1c and diabetic retinopathy is expected to differ between different races. This study was designed to verify whether HbA1c or fasting plasma glucose (FPG) is more effective in detecting diabetic retinopathy in a Japanese population.

**Materials and Methods:**

The study subjects underwent health examinations between 2008 and 2009 with fasting. Of these participants, we analyzed the data for 2,921 Japanese men who had undergone an ophthalmologic examination. Retinopathy was classified into 7 categories according to a simplified diabetic retinopathy scale. The odds ratios of retinopathy according to the eight groups of FPG and HbA1c were estimated using multiple logistic regression analysis adjusted for age. Receiver operator characteristic analysis was used to evaluate each value associated with the presence or absence of retinopathy. *Results and Discussion*. The odds ratios (95% CI) of retinopathy for HbA1c level categories, in ascending order, were 1.0 (ref.), 0.88 (0.28-2.75), 1.27 (0.44-3.69), 1.52 (0.48-4.79), 1.89 (0.52-6.85), 2.70 (0.66-11.10), 4.10 (0.80-21.00), and 6.34 (2.37-16.97) where the odds ratios significantly increased with HbA1c ≥ 6.8%. The area under the curve (SE) for FPG and HbA1c was almost the same, at 0.668 (0.043) and 0.680 (0.043), respectively.

**Conclusions:**

It was clarified that the higher the level of HbA1c, the higher the prevalence of retinopathy, and there was no clear threshold. The detection ability of retinopathy was almost the same, suggesting that it is possible to detect the risk of retinopathy by HbA1c only.

## 1. Introduction

In recent years, lifestyle-related diseases such as diabetes have become important issues in developed countries as well as developing countries, with the dramatic transformation of lifestyles due to economic development [1]. The annual report of the World Health Organization in 2002 warned that the number of diabetic patients would increase dramatically, especially in developing countries of Asia, during the next thirty years. It is obvious that preventive measures for diabetes should be implemented early.

Diabetes causes three major complications: retinopathy, nephropathy, and neuropathy. Diabetes also raises the risk of cardiovascular disease. Of the three major complications of diabetes, retinopathy appears relatively early. In Japan, the National Health and Nutrition Survey 2007 reported that 10.6% of the population aged 20 or above who were diagnosed with diabetes had diabetic retinopathy [2].

To diagnose diabetes, fasting blood glucose levels are measured twice on different days. The international advisory committee, assembled by the American Diabetes Association (ADA), the International Diabetes Federation (IDF), and the European Association for the Study of Diabetes (EASD), recommended one measurement of HbA1c be used as a new diagnostic criterion for diabetes (announced June 5, 2009, at the symposium of the 69^th^ American Diabetes Association). Diabetes is currently diagnosed using fasting blood glucose levels and oral glucose tolerance tests in western countries and Japan. HbA1c is used as an index to reflect the average blood glucose levels of the past 1-2 months. A cross-sectional study, in which the relationship between HbA1c and retinopathy was observed in 960 Pima Indians, 1,018 Egyptians, and 2,821 Americans, reported that the risk of the onset of diabetes was high when HbA1c was 6.0% or more and less than 6.5% [3]. Based on this report, the committee commented that HbA1c testing was also useful for the detection of a prediabetic group, and although “6.5% should not be an absolute threshold,” “people whose HbA1c level is getting close to 6.5% will greatly benefit by taking measures to prevent diabetes.” The relationship between HbA1c and diabetic retinopathy is expected to differ based on race, but the HbA1c threshold has not yet been determined in Japanese subjects.

In this study, we verified which of the measured values, HbA1c or fasting plasma glucose (FPG), is more effective in detecting diabetic retinopathy in a Japanese population.

## 2. Materials and Methods

A total of 9,662 male employees aged 45 or over underwent an annual health check-up after having fasted overnight. All the examinations were performed between 2008 and 2009 in Hitachi, Ibaraki prefecture. Among the participants, 4,582 subjects had detailed clinical data related to diabetes. Most (4,133) had fundus photographs; however, participants were excluded if the photographs were not clear enough due to cataracts, corneal opacity, or other reasons; 2,921 participants were included in the final study. Both nondiabetes and diabetes patients at the baseline were included as participants. Retinopathy was assessed with single-field 45° nonmydriatic fundus photography of each eye using a digital fundus camera (TRC NW300, Topcon Inc., Japan). Retinopathy was classified into 7 categories, according to the International Clinical Diabetic Retinopathy Disease Severity Scale [4]. The categories were (1) no apparent retinopathy, (2) mild nonproliferative diabetic retinopathy, (3) moderate nonproliferative diabetic retinopathy, (4) severe nonproliferative diabetic retinopathy, (5) proliferative diabetic retinopathy, (6) light coagulation, and (7) after surgery. We defined subjects with (2) to (7) as having diabetic retinopathy. To avoid diagnostic bias [5, 6], assessments were performed without prior knowledge of FPG and HbA1c levels and were based on independent grading of the worst eye by two ophthalmologists specialized in retinal disease. The two ophthalmologists independently examined the fundus images. If the opinions of the two ophthalmologists differed, then the ophthalmologists would discuss the results again to reach an agreement. Patients with obvious differential diagnosis, such as retinal vein occlusion, were judged to have no diabetic retinopathy. Participants with cataracts and corneal opacity were excluded because their fundus photography was unclear and difficult to assess.

Height and weight were measured using an automated scale (BF-220; Tanita, Tokyo, Japan) with the patient wearing a light gown. BMI was calculated using the weight (kg) divided by the square of the height (m). Blood was collected from each subject after more than 12 h of fasting. Blood glucose levels were measured using the glucose electrode technique with an ADAMS glucose GA1170 device (Arkrey). HbA1c levels were measured using a HPLC method with an ADAMS HA8160 device (Arkrey). Blood pressure was measured using an oscillometric method with a Kentaro ADVANCEBP-203RV III A/B device (Colin) while the patient was in a sitting position and after the patient had rested for 3 min. Informed consent was obtained from each subject regarding the use of his or her data for research purposes. The present study protocol was approved by the ethics review committee of the National Center for Global Health and Medicine (Tokyo, Japan).

FPG and HbA1c levels were categorized into eight groups: Group 1, ≤94 mg/dl; Group 2, 95-99 mg/dl; Group 3,100-104 mg/dl; Group 4, 105-109 mg/dl; Group 5, 110-117 mg/dl; Group 6, 118-125 mg/dl; Group 7, 126-144 mg/dl; and Group 8, ≥145 mg/dl for FPG and Group 1, ≤5.5%; Group 2, 5.6-5.7%; Group 3, 5.8-5.9%; Group 4, 6.0-6.1%; Group 5, 6.2-6.3%; Group 6, 6.4-6.5%; Group 7, 6.6-6.7%; and Group 8, ≥6.8% for HbA1c. The numbers of participants in each of the eight groups of FPG and HbA1c levels are shown in Table 1. The classification of fasting plasma glucose at 100, 110, and 126 mg/dl is based on clinical criteria. Otherwise, we divided them into groups to avoid disproportionate numbers. Participants in the HbA1c group were divided into groups of 0.2% increments at even intervals in order to explore a potential threshold.

The prevalence of retinopathy in the eight groups of FPG and HbA1c is shown in Table 1. The clinical characteristics of the subjects among the eight groups of FPG and HbA1c groups were compared in terms of age-adjusted means calculated by analysis of covariance (ANCOVA). *P* values were adjusted for multiple comparisons using Bonferroni-Holm's method. Odds ratios (95% confidence intervals (CI)) to detect retinopathy according to the eight groups of FPG and HbA1c were estimated using multiple logistic regression analysis adjusted for sex and age.

Receiver operator characteristic (ROC) analysis was used to evaluate each level of FPG and HbA1c associated with the presence of retinopathy. ROC analysis is a formal method that plots sensitivity against 1-specificity to assess the trade-off between sensitivity and specificity at various test cut-off points or thresholds, providing a measure of diagnostic accuracy called the area under the curve (AUC). We drew the ROC curve for each FPG and HbA1c and calculated the corresponding AUC. All analyses were performed using SPSS for Windows version 15.0 (SPSS, Chicago, IL) and Stata 10 (Stata LP, College Station, TX).

## 3. Results

Comparisons of the clinical characteristics of subjects in the FPG and HbA1c groups are shown in Table 1. The higher the FPG, the higher the blood pressures and BMI. The higher the HbA1c, the higher the BMI. The number of patients with retinopathy was 49 out of 2,921 participants (1.7%). The number of patients who are regularly visiting the hospital for treatment of diabetes was 170 out of 2,921 participants (5.8%).

**Figure 1:**
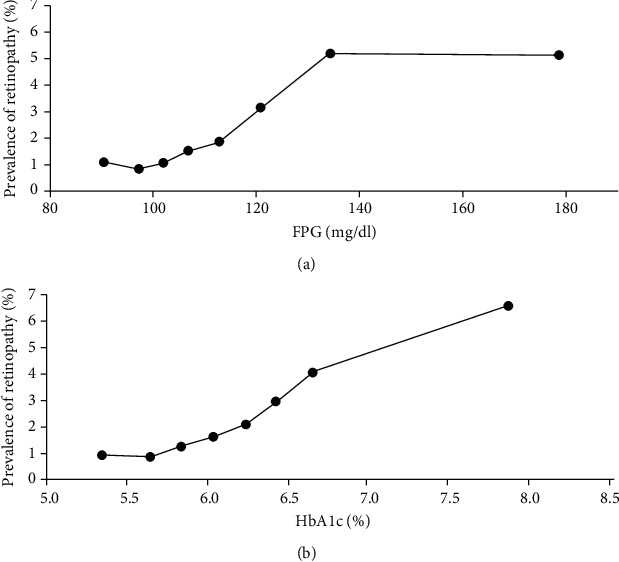
(a) Prevalence of retinopathy according to the levels of FPG. The *x*-axis shows the average of each group. (b) Prevalence of retinopathy according to the levels of HbA1c. The *x*-axis shows the average of each group.

**Figure 2:**
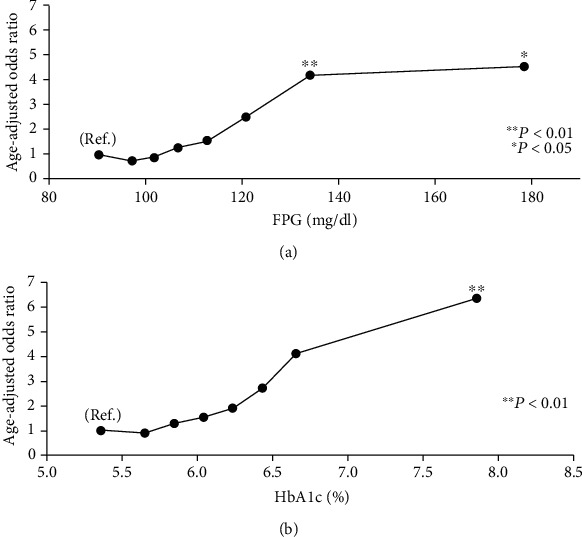
(a) The age-adjusted odds ratio of retinopathy according to the levels of FPG. The *x*-axis shows the average of each group. (b) The age-adjusted odds ratio of retinopathy according to the levels of HbA1c. The *x*-axis shows the average of each group.

The prevalence of retinopathy is shown by FPG levels in Figure 1(a) and by HbA1c levels in Figure 1(b). It was shown that the higher the levels of FPG and HbA1c were, the higher the prevalence of retinopathy is. The age-adjusted odds ratios for retinopathy according to the FPG and HbA1c levels are shown in Figures 2(a) and 2(b). The age-adjusted odds ratios (95% CIs) for the prevalence of retinopathy in the FPG categories were 1.0 (ref.), 0.75 (0.23-2.46), 0.90 (0.29-2.83), 1.28 (0.41-4.02), 1.53 (0.51-4.60), 2.51 (0.75-8.37), 4.18 (1.42-12.32), and 4.54 (1.43-14.36). The age-adjusted odds ratios of the prevalence of retinopathy in the HbA1c categories were 1.0 (ref.), 0.88 (0.28-2.75), 1.27 (0.44-3.69), 1.52 (0.48-4.79), 1.89 (0.52-6.85), 2.70 (0.66-11.10), 4.10 (0.80-21.00), and 6.34 (2.37-16.97). The prevalence and odds ratios for retinopathy increased with increasing FPG and HbA1c levels. The results suggest that the odds ratios markedly increase in the top two level categories of FPG and HbA1c. To determine whether there are threshold levels of FPG and HbA1c in the development of retinopathy, the odds ratios adjusted for age were plotted on a logarithmic scale. They showed that for both FPG and HbA1c levels, the odds ratio for the development of retinopathy increased almost linearly with the increases in the levels of FPG and HbA1c, and no clear threshold was observed (data not shown). The AUC values (SE) of the ROC curve for FPG and HbA1c to detect retinopathy were almost the same, at 0.668 (0.043) and 0.680 (0.043), respectively.

## 4. Discussion

We compared the ability to detect retinopathy between levels of FPG and HbA1c: both FPG and HbA1c had almost the same predictive ability for detecting retinopathy.

Previous cross-sectional studies to detect the optimal cut-off values of HbA1c have been reported. A population-based sample of Malay adults demonstrated that the optimal HbA1c cut-off point for detecting mild and moderate retinopathy was between 6.6 and 7.0% [7]. In the Ansung Cohort Study, the prevalence of diabetic retinopathy was 1.6%, although for moderate or severe retinopathy it was 0.5% when HbA1c levels were 6.5-6.8% [8]. A data pooling analysis of five studies from five countries showed that the prevalence of moderate or severe diabetic retinopathy was 2.6% when HbA1c levels were 6.5% [9]. The prevalence of retinopathy was 1.7% in our study which may be lower than the prevalence in the general population because of the healthy worker effect. The prevalence and the odds ratio for retinopathy increased as the HbA1c level increased. When HbA1c was ≥6.8%, the odds ratio for retinopathy was significantly increased. Since Japanese have a lower insulin secretory capacity than Caucasians, they are more likely to have diabetes. Therefore, we suggest that there may be racial differences in the susceptibility to diabetic retinopathy. The prevalence of retinopathy was 6.6% when HbA1c levels were ≥6.8% and the age-adjusted odds ratio for the group was 6.34 using HbA1c ≤ 5.5% as the reference. Patients whose HbA1c levels were close to 6.8% were at a high risk of retinopathy. Considering that this study was a cross-sectional study, it was suggested that the progression of retinopathy started from a stage with somewhat lower HbA1c levels; therefore, health interventions should be started from an earlier phase.

Several studies have compared the sensitivity of FPG and HbA1c in diabetic retinopathy detection. Many cross-sectional studies conducted in China, Iran, Korea, the US, Pima peoples, etc., have reported that HbA1c was able to detect diabetic retinopathy more sensitively than FPG [8, 10–12]. A meta-analysis also indicated the superiority of HbA1c to FPG in detecting diabetic retinopathy: the odds ratio was 16.32 for HbA1c and 4.87 for FPG, and the area under the ROC curve was 0.837 for HbA1c and 0.735 for FPG [13]. In contrast, there are only two studies which report that FPG is the superior indicator when compared with HbA1c [14, 15].

Several studies report on the optimal cut-off values for HbA1c; however, it is challenging to compare the values between studies due to methodological inconsistencies, including sampling procedures: nondiabetes, diabetes, or both; the conditions under which the fundus photographs are taken; and diagnostic criteria for diabetic retinopathy [8, 10–12, 14–23]. Nevertheless, three studies have examined the cut-off values of HbA1c using the same criteria for participants and diagnostic methods for fundus images, that is, both nondiabetes and diabetes patients and the international clinical disease severity scale for retinopathy used in our study. One study involving 3,403 Chinese patients found that the optimal cut-off value of HbA1c to detect diabetic retinopathy was 6.6%, and for those with moderate or severe retinopathy, it was 6.9% [8]. Another study examining 2,551 Chinese patients reported that the prevalence of diabetic retinopathy significantly increased in patients with HbA1c levels of 6.4% or higher [22]. A study of 3,010 Iranian patients reported that the optimal cut-off point of HbA1c was 6.2% [10]. Our study revealed that the odds ratio of retinopathy significantly increased when the HbA1c level exceeded 6.8%, which is slightly higher than the values reported by the other studies.

This study has several strengths and limitations. One of its strengths is the sample size which was sufficiently large (2,921 subjects). Second, to avoid the bias in diagnosis, we used two independent ophthalmologists who specialized in retinal disease and had no prior knowledge of the participants' FPG or HbA1c levels. The limitations of this study include the use of single-field retinal photography for measuring retinopathy; however, a comparison of 7-fold standard stereoscopic fundus photographs, single-field standard stereoscopic fundus photographs, and single-field nonmydriatic retinal photography showed perfect agreement in identifying people with and without diabetic retinopathy and moderate agreement for retinopathy grading [24]. In addition, because of its cross-sectional design, changes or incidence of retinopathy were not monitored. It was not possible to examine the different degrees of retinopathy due to the size of the study; however, in the future, we would like to accumulate enough data for a longitudinal study, analyzing the different degrees of retinopathy.

## 5. Conclusions

We clarified that the higher the level of HbA1c, the higher the prevalence of retinopathy, and there was no clear threshold. We compared the ability to detect retinopathy between FPG and HbA1c using ROC curves: the detection ability was almost the same, suggesting that it is possible to detect the risk of retinopathy using HbA1c only. In order to prevent retinopathy, it is clear that HbA1c should remain at a low level, within a range of not causing hypoglycemia. Further prospective studies are needed to assess the impact of HbA1c levels on the incidence of diabetic retinopathy.

## Figures and Tables

**Table 1:** Comparison of the clinical characteristics of the subjects in the FPG and HbA1c groups (*n* = 2,921).

FPG		Number with retinopathy/total	Systolic blood pressure (mmHg)			Diastolic blood pressure (mmHg)			BMI (kg/m^2^)		
			Mean	SE	*P* value	Mean	SE	*P* value	Mean	SE	*P* value
Group 1	≤94 mg/dl	6/540 (1.1%)	119.1	(0.5)	Ref.	76.4	(0.3)	Ref.	22.9	(0.1)	Ref.
Group 2	95-99 mg/dl	5/595 (0.8%)	121.0	(0.5)	0.006	78.2	(0.3)	0.0003	23.6	(0.1)	<0.0001
Group 3	100-104 mg/dl	6/574 (1.0%)	121.9	(0.5)	0.0001	77.9	(0.3)	0.003	24.1	(0.1)	<0.0001
Group 4	105-109 mg/dl	6/399 (1.5%)	123.7	(0.6)	<0.0001	79.4	(0.4)	<0.0001	24.2	(0.1)	<0.0001
Group 5	110-117 mg/dl	7/381 (1.8%)	125.4	(0.6)	<0.0001	80.1	(0.4)	<0.0001	24.6	(0.1)	<0.0001
Group 6	118-125 mg/dl	5/161 (3.1%)	126.1	(0.6)	<0.0001	80.5	(0.6)	<0.0001	25.2	(0.2)	<0.0001
Group 7	126-144 mg/dl	8/154 (5.2%)	123.9	(0.9)	<0.0001	78.7	(0.6)	0.003	24.9	(0.2)	<0.0001
Group 8	≥145 mg/dl	6/117 (5.1%)	125.8	(1.0)	<0.0001	78.5	(0.7)	0.006	24.7	(0.2)	<0.0001

HbA1c		Number with retinopathy/total	Systolic blood pressure (mmHg)			Diastolic blood pressure (mmHg)			BMI (kg/m^2^)		
			Mean	SE	*P* value	Mean	SE	*P* value	Mean	SE	*P* value
Group 1	≤5.5%	6/657 (0.9%)	122.9	(0.5)	Ref.	79.1	(0.3)	Ref.	23.1	(0.1)	Ref.
Group 2	5.6-5.7%	6/708 (0.8%)	121.2	(0.5)	0.091	77.7	(0.3)	0.005	23.6	(0.1)	0.001
Group 3	5.8-5.9%	8/629 (1.3%)	122.1	(0.5)	1.0	78.5	(0.3)	0.67	24.1	(0.1)	<0.0001
Group 4	6.0-6.1%	6/372 (1.6%)	122.3	(0.6)	1.0	78.2	(0.4)	0.34	24.5	(0.1)	<0.0001
Group 5	6.2-6.3%	4/192 (2.1%)	124.1	(0.9)	1.0	78.8	(0.6)	1.0	25.1	(0.2)	<0.0001
Group 6	6.4-6.5%	3/101 (3.0%)	124.0	(1.2)	1.0	78.6	(0.8)	1.0	24.6	(0.3)	<0.0001
Group 7	6.6-6.7%	2/49 (4.1%)	122.8	(1.7)	1.0	78.7	(1.1)	1.0	25.3	(0.4)	<0.0001
Group 8	≥6.8%	14/213 (6.6%)	123.4	(0.8)	1.0	77.8	(0.5)	0.18	24.8	(0.2)	<0.0001

Mean and standard error (SE) were adjusted for age by analysis of covariance (ANCOVA). *P* values were for comparisons between Group 1 and other groups and adjusted for multiplicity (7 comparisons) using Bonferroni-Holm's method.

## Data Availability

The data used to support the findings of this study are available from the corresponding author upon request.
